# S100 Proteins—Intracellular and Extracellular Function in Norm and Pathology

**DOI:** 10.3390/biom14040432

**Published:** 2024-04-02

**Authors:** Wiesława Leśniak, Anna Filipek

**Affiliations:** Nencki Institute of Experimental Biology, Laboratory of Calcium Binding Proteins, 3 Pasteur Street, 02-093 Warsaw, Poland; a.filipek@nencki.edu.pl

## 1. Introduction

The S100 proteins are small, ubiquitous, mostly homodimeric proteins containing two EF-hand structures, that is, helix-loop-helix motifs specialized in high-affinity calcium-binding (~10^−6^ M) [1]. In resting cells, S100 proteins exist in an inactive, close conformation, but upon calcium influx most of them undergo a spectacular conformational change manifested by the exposure of hydrophobic surfaces that enable them to interact with various protein ligands [2]. They can thus be considered “readers” of the calcium signal that transmit the external stimulus to various intracellular effectors. Many such effectors have been identified throughout the years. They include, among others, cytoskeletal proteins, transcription factors, receptors, and enzymes such as kinases and phosphatases. Some S100 proteins can also be secreted and act outside the cell by binding to various receptors or their ligands. Despite sequence homology (25–65%) and structural similarity, each of the more than twenty S100 protein family members has unique features that largely determine the scope and type of interacting proteins [1]. This, coupled with different expression patterns or cellular localization of particular S100 proteins, makes the spectrum of their interactions even broader and more diversified, with many unique as well as common targets. Consequently, the S100 proteins play multiple, non-redundant functions in the cell. They are involved in cell adhesion, motility, apoptosis, proliferation, differentiation, and other processes. On a wider scale, they contribute to cellular stress response, inflammatory processes, carcinogenesis, neurodegeneration, etc. [3,4,5,6,7]. It is therefore not surprising that multiple S100 proteins are linked to various human pathologies [1]. This association is based on the fact that expression of particular S100 proteins is altered in multiple diseases and that modification of their levels, accomplished in in vitro or in vivo experiments, can affect the molecular and clinical parameters or even the outcome of a given disease [8]. Therefore, there is much hope that further research will help to establish the causative link between S100 proteins and various diseases and, in a further perspective, bring some therapeutic advantages.

## 2. An Overview of Published Articles

The main challenge in S100 protein research is thus to find out how the sum of their interactions with various targets translates into the development and/or progression of different pathologies. To achieve this goal, the ongoing research on S100 proteins aims to identify the full repertoire of their ligands as well as resolve subtle details of their structure, especially in the context of ligand binding. The search for agents that can interfere with ligand binding is also underway and may prove valuable for designing S100 protein-targeted therapies. Many studies are devoted to the role of particular S100 proteins in specific cellular processes, especially those that are characteristic of or become dysregulated under pathological conditions. Also, there are multiple studies concerning regulation of their expression and possible correlations between the expression of particular S100 proteins and various diseases.

The 15 articles published in the two editions of the Special Issue of *Biomolecules* entitled “S100 Proteins—Intracellular and Extracellular Function in Norm and Pathology” aptly illustrate the state of the art and the current trends in S100 protein research. Several contributions (e.g., 4, 5, and 9) explore the universe of novel or established S100 protein interactions and ways to inhibit them, or even, as in the case of contribution 12, present a strategy for inhibitor-dependent S100 protein degradation. Other contributions (e.g., 1, 2, 3, 6, and 10) examine the expression or involvement of particular S100 proteins in processes such as cell motility, proliferation, and inflammation. Still others look for possible correlations between the level of particular S100 proteins and various pathologies (e.g., 8, 11, and 12) in order to establish them as disease markers or provide comprehensive reviews on S100 protein function in norm and pathology (e.g., 7, 14, and 15).

**Contributions 1** and **10** (Ismail et al., 2021; Lancaster et al., 2023) explore the role of the S100P protein in cell motility in the context of cancer metastasis (**1**) and extravillous trophoblast motility and invasion during pregnancy (**10**) and reveal two independent mechanisms of S100P action. While the intracellular protein enhances cell motility through interaction with myosin IIA and modulation of the focal adhesion pathway, the extracellular, membrane-bound protein acts by a yet unknown mechanism that does not involve changes in focal adhesion.

**Contribution 2** (Drosatos et al., 2021) refers to the role of the S100A8/S100A9 heterodimer in inflammatory processes and shows that physical exercise is effective in lowering S100A8, S100A9, and soluble RAGE levels concomitantly with lower proinflammatory cytokine production.

**Contribution 3** (Głowacka et al., 2021) offers an overview of the stimuli inducing S100A10 expression and reveals that the GRHL2 transcription factor is involved in S100A10 gene transcription by binding within its intronic region.

**Contribution 4** (Katte et al., 2021) employs the NMR technique to examine in detail the interaction between S100P and the N-terminus of p53 and shows that pentamide disrupts this interaction to the effect that it can reactivate p53 and block proliferation of breast cancer cells.

**Contribution 5** (Yamamoto et al., 2021) identifies a novel interaction between S100A6 (and some other S100 proteins) and the high mobility group HMG20A protein and maps the site of S100A6 binding.

**Contribution 6** (Garcia et al., 2022) presents an interesting overview of the role of the S100A8/S100A9 heterodimer, also known as calprotectin, in inflammation. The article concentrates on the binding of calprotectin to various cellular receptors mediating inflammatory responses as well as on agents that inhibit those interactions, critically assessing their specificity and potential therapeutic use. 

**Contributions 7** and **14** (Bharadwaj et al., 2021 and Okura et al., 2023) are review articles devoted to S100A10, also known as p11, an atypical S100 protein that does not bind calcium and which, through its interaction with annexin A2 (Anx2), plays an essential role in plasmin generation and, consequently, in fibrinolysis. Contribution **7** deals with a very interesting aspect of complex formation between the S100A10 protein and Anx2, that is, the acquisition of unique properties that could not be attributed to either of the two components of the complex. We come to know how the localization, expression, stability, and function of these two proteins are changed in the presence of the other one. Contribution **14** gives a more general picture of the structure and multiple interactions of S100A10 and concentrates on recent advances in the field. 

**Contributions 8** and **13** (Tsoporis et al., 2023; Gkavogiannakis et al., 2023) evaluate the suitability of assessing the plasma level of certain S100 proteins (and other DAMPs) as markers of various human pathologies, such as coronary artery ectasia or asthma. Results show that specific S100 proteins may indeed serve as markers of these pathologies and even help to differentiate disease subtypes.

**Contribution 9** (Kazakov et al., 2023) identifies novel ligands of the S100A6 protein among the family of four-helical cytokines and maps a cytokine-binding site within the S100A6 molecule. The findings indicate that S100A6 is able to interfere with cytokine signaling and that, prospectively, this property can be used to attenuate the effect of excessive cytokine release that is common to many disorders.

**Contribution 11** (Mandarino et al., 2023) denotes correlations between the expression of various S100 proteins and between S100 proteins and the high mobility group box 1 (HMGB1) protein in pancreatic cancer cell lines and tissues. Such correlations point to common elements involved in the regulation of their expression, among them the RAGE receptor. 

**Contribution 12** (Ismail et al., 2023) presents and examines a novel strategy to reduce the rise in the level of the S100A4 protein, an S100 family member with strong involvement in cell motility and invasion and therefore associated with cancer metastases, by chemically coupling its inhibitor to an agent that directs the protein-inhibitor complex to proteasome-mediated destruction. The results show that this strategy was effective in a triple-negative breast cancer mouse model.

**Contribution 15** (Basnet et al., 2023) offers a comprehensive updated review of the S100A16 protein in various human cancers. Starting with information about the structure and normal expression pattern of S100A16, the review proceeds to present available data on the expression, interaction network, and influence of S100A16 on cell proliferation, motility, invasiveness, and other cancer-related cellular features observed in various malignancies. 

## 3. Conclusions

The S100 proteins are a group of structurally related small calcium-binding proteins that can interact with and alter the activity or function of multiple ligands. The ability to interfere with many signaling pathways as well as the correlation between the level of individual S100 proteins and the progression of various diseases argue for their involvement in many pathologies [1]. As of now, numerous S100 proteins have been proposed as markers of certain diseases that cannot only facilitate diagnosis but also be instructive as to the stage of the disease and patient prognosis [1,8]. Since many S100 proteins are active extracellularly and are easily detectable in blood and other body fluids, their use as disease markers may soon enter the clinic. On the other hand, the ongoing search for specific and effective inhibitors that disturb S100 protein interactions with various targets, coupled with studies on the structure and conformation of S100-ligand or S100-inhibitor complexes, may one day prove useful in designing S100-targeted therapies [9]. Certainly, progress in S100 protein research should soon fill the gap between scientific observations and therapeutic approaches to the advantage of patients. 
